# Improving skills and care standards in the support workforce for older people: a realist synthesis of workforce development interventions

**DOI:** 10.1136/bmjopen-2016-011964

**Published:** 2016-08-25

**Authors:** L Williams, J Rycroft-Malone, C R Burton, S Edwards, D Fisher, B Hall, B McCormack, S M Nutley, D Seddon, R Williams

**Affiliations:** 1 School of Healthcare Sciences, Bangor University, Bangor, UK; 2 Queen Margaret University, Edinburgh, UK; 3 University of St Andrews, St Andrews, UK

**Keywords:** EDUCATION & TRAINING (see Medical Education & Training)

## Abstract

**Objectives:**

This evidence review was conducted to understand how and why workforce development interventions can improve the skills and care standards of support workers in older people's services.

**Design:**

Following recognised realist synthesis principles, the review was completed by (1) development of an initial programme theory; (2) retrieval, review and synthesis of evidence relating to interventions designed to develop the support workforce; (3) ‘testing out’ the synthesis findings to refine the programme theories, and establish their practical relevance/potential for implementation through stakeholder interviews; and (4) forming actionable recommendations.

**Participants:**

Stakeholders who represented services, commissioners and older people were involved in workshops in an advisory capacity, and 10 participants were interviewed during the theory refinement process.

**Results:**

Eight context–mechanism–outcome (CMO) configurations were identified which cumulatively comprise a new programme theory about ‘what works’ to support workforce development in older people's services. The CMOs indicate that the design and delivery of workforce development includes how to make it real to the work of those delivering support to older people; the individual support worker's personal starting points and expectations of the role; how to tap into support workers' motivations; the use of incentivisation; joining things up around workforce development; getting the right mix of people engaged in the design and delivery of workforce development programmes/interventions; taking a planned approach to workforce development, and the ways in which components of interventions reinforce one another, increasing the potential for impacts to embed and spread across organisations.

**Conclusions:**

It is important to take a tailored approach to the design and delivery of workforce development that is mindful of the needs of older people, support workers, health and social care services and the employing organisations within which workforce development operates. Workforce development interventions need to balance the technical, professional and emotional aspects of care.

**Trial registration number:**

CRD42013006283.

**Strengths and limitations of this study**

- Applying a novel methodological approach enabled a theory-driven explanation of how workforce development for support workers can be successful.
- The process of the review facilitated the development of a new programme theory, which can be used to guide workforce development initiatives in the future.
- The use of an embedded approach to stakeholder engagement promoted joint decision-making at key stages in the study process.
- The extent of evidence to support some elements of the programme theory was limited at times, especially as reports of interventions lacked specificity.

## Background

In the context of an ageing population and high profile reviews about the quality of health and social care services provision for older people, there is a pressing need to focus on workforce development for National Health Service (NHS) and social care staff who provide care,^1^ including support workers.^2^ Support workers provide ‘face to face care or support of a personal or confidential nature to service users in clinical or therapeutic settings, community facilities or domiciliary settings, but who do not hold qualifications accredited by a professional association, and are not formally regulated by a statutory body’.^3^ Across health and social care services, the UK support workforce represents an estimated 1.3 million individuals working in practice.^4^ Support workers have varied roles that have been described under four domains,^5^ including direct care (where the support worker works directly with the individual), indirect care (undertaken to support a plan of care), administration (does not involve direct contact with the individual) and facilitation (to support the team or environment in which the support worker is working). The evidence shows that support workers often feel undervalued within their employing organisation despite taking on more skilled work,^3^ and they also feel unsupported to develop clear career pathways.^6^ ^7^

Further evidence to inform older people's services about how to improve care standards is important, especially in the light of the introduction of new service models (eg, integrated services), where the support worker can be expected to work with different organisations and across traditional boundaries.^8^ This review addresses a gap in knowledge by providing a theory-driven, synthesised account of the evidence for developing the support workforce. The working definition of workforce development interventions used for the review was the support required to equip those providing care to older people with the right skills, knowledge and behaviours to deliver safe and high quality services.^9^

### Research question

How can workforce development interventions improve skills and the care standards of support workers within older people's health and social care services?

### Aims

The aims of the study were to:

1. Identify evidence about support worker development interventions from different public services and synthesise evidence of impact.
2. Identify the mechanisms through which these interventions deliver support workforce and organisational improvements that are likely to benefit the care of older people.
3. Investigate the contextual characteristics that mediate the potential impact of these mechanisms on care standards for older people.
4. Develop a practical programme theory from the evidence that synthesises findings of relevance for services delivering care to older people.
5. Recommend improvements for the design and implementation of workforce development interventions for support workers.

## Methods

We recognised that workforce development for the support workforce for older people's care services is complex, involving various people, structures and organisations, and its effectiveness is contingent upon a variety of factors.^10^ Therefore, the study was designed using an approach that could accommodate complexity and contingency.^10^ We undertook a realist synthesis underpinned by a realist philosophy of science and causality.^11^ ^12^ In realist synthesis, contingent relationships are expressed as context–mechanisms–outcome (CMO) configurations, to show how particular contexts or conditions trigger mechanisms to generate certain outcomes. In realist terms, programme theory ‘describes the theory built into every programme’,^13^ and it is the interaction between the unseen elements of a programme (the mechanisms), with particular condition or contextual factors which explains the outcomes that result from the programme interventions. Mechanisms are the ‘causal forces or powers’ that lead to outcomes.^14^ The programme theory may also show how the CMO configurations are inter-related, to illuminate how the coveted programme outcomes can be achieved.

Reflecting the importance of stakeholder engagement in realist reviews, we linked with a number of managers, nurses, educators, commissioners and older people's representatives in elaborating the study context, refining the review questions, contributing to programme theory development and interpreting the evidence. The RAMESES publication standards were used to guide this report.^12^

### Changes to the review process

No changes to the review process were made subsequent to the publication of the review protocol (http://bmjopen.bmj.com/content/4/5/e005356.full).

The study was conducted in four phases.

### Phase I

Concept mining was undertaken to map evidence about the support workforce, workforce development interventions, older people's services, how interventions might operate and any reported enablers or barriers to the successful implementation of interventions. Concept mining in realist synthesis describes a process of searching through different bodies of evidence for information that could help build theories. In this review, concept mining involved searching through different bodies of evidence (including the commissioning brief, policy/guidance and grey literature) for information that could build theories about workforce development. For example, from policy documents, we found evidence relating to perceptions about support worker roles, gaps identified in skills training, ideas about how training and development should be structured for the support worker and suggested approaches to workforce development, and literature relating to professionalism and the working environment.

We conducted a workshop in which stakeholders contributed to developing the scope of the study and building the initial programme theories. The structure of the theory-building workshop was guided by soft systems thinking, a learning approach that offers an interpretive view of the complex and adaptive nature of human systems within the ‘real world’.^15^ ^16^ Soft systems thinking also enabled the generation of rich pictures describing how workforce development works. An extensive list of issues and related questions in four theory areas was generated by the review team, drawn from evidence and stakeholders' perspectives, which were subsequently reviewed and prioritised by the workshop participants and then by the study's Advisory Group members in a face-to-face meeting (see online supplementary additional file 1).

10.1136/bmjopen-2016-011964.supp1 Supplementary additional file

### Phase II

#### Search strategy

We developed a comprehensive search strategy, led by the project's information scientist and involving the research team and feedback from the steering group, and supplemented a primary search with purposive searches in order to capture the most relevant evidence to support or refute the theories. As an iterative process, searching became more focused as the review progressed and theories were refined. Specific search terms for support workers in education and policing were also used to identify any cross-sector learning from the existence of support roles in these public service areas. Major health, social care and welfare databases were searched using selected generic keywords and database-specific keywords. The primary search was limited to material from 1986 to 2013 to reflect the period after the conception of National Vocational Qualifications (NVQ) qualifications for support workers. Methodological filters were not used to avoid excluding any potentially relevant articles. Systematic searches were conducted in 11 electronic databases. These were PsycINFO, Health Technology Assessment, Social Services Abstracts, Sociological Abstracts, MEDLINE, NHS Economic Evaluation Database, Web of Science, CINAHL, COCHRANE, Applied Social Sciences Index and Abstracts and Database of Abstracts & Reviews of Effects. The searches took place in April–May 2014. References were stored in Ref Works. The database search yielded 17 033 references, of which 4684 were duplicates leaving 12 349 hits included for title screening (see online supplementary additional file 2). Alerts were set up for ongoing database searches and these alerts were scanned up to April 2015.

10.1136/bmjopen-2016-011964.supp2 Supplementary additional file

The purposive searching, which has been found to be a useful strategy in realist synthesis, included searches for support worker role evaluations, and intervention research that made specific reference to embedded implementation or impact (eg, around careers, location, settings, skills and outcomes). Purposive searches were conducted in AMED, HMIC, education, policing and the health-related practice development literature. Hand searching was conducted in the *British Journal of Healthcare Assistants (BJHCA)*. The logic for additionally looking beyond health and social care (education and policing) was to seek cross-sector learning given that support roles exist in other public services and there is potential transferability of good practice. Other articles were added through snowballing, from database alerts and from suggestions by stakeholders, including the advisory group members and workshop attendees. Additionally, internet-based searches for grey literature were conducted for workforce development project reports—national inspection and regulation quality reports.

#### Selection and appraisal of documents

Following realist synthesis principles, the test for inclusion was evidence that was good enough and relevant.^17^ However, we consider that the test of good enough and relevant is potentially vague which could lead to a lack of transparency about decision-making. In this review, using critical discussion within the core team, we developed an additional set of constructs to sit alongside data extraction forms, which deconstructed the test as fidelity (faithfulness or match with the initial programme theories), trustworthiness (that the evidence can be relied upon), ‘nuggets’ (valuable data) and relevance (the contribution of the evidence to the review) (see online supplementary additional file 3). Member checking of the review process took place within the research team. Title-sifting was cross-checked across three team members (JR-M, CRB and LW). Levels of agreement across reviewers were scored for 6% of the total titles. The title-sifting example was also checked with JR-M, CRB, LW and BH. The quality and relevance of the evidence was assessed during the synthesis process through weighing up the contribution of data to the development of the study's explanatory account, review question and aims.

10.1136/bmjopen-2016-011964.supp3 Supplementary additional file

### Phase III

Theory development, refinement and testing were iterative processes made visible through bespoke data extraction forms developed from the four theory areas generated in phase I, to provide a template to extract evidence. Data were organised into evidence tables representing the four theory areas (eg, see online supplementary additional file 4 (Theory area 1)). As data were extracted, we also began the process of synthesis. The realist synthesis is theory-driven, and abductive reasoning was used to understand CMO configurations.^18^ We used abduction (ie, seeing something new in evidence or observation and making inference to the plausible explanations about the cause) and retroduction (ie, understanding the cause of an event beyond what can be seen), checking and prioritising across the evidence tables to look for emerging patterns (eg, see online supplementary additional file 5). This process was facilitated by the development of a set of plausible hypotheses: ‘if…then’ statements about what might work, for whom, how, why and in what circumstances (related to workforce development interventions for the support care workforce) (see online supplementary additional file 6). Plausible hypotheses evidence tables were then used as the basis for further deliberations between the core group and stakeholders about the contingent threads emerging from the analysis of the evidence base, that is, the eight CMOs.

10.1136/bmjopen-2016-011964.supp4 Supplementary additional file

10.1136/bmjopen-2016-011964.supp5 Supplementary additional file

10.1136/bmjopen-2016-011964.supp6 Supplementary additional file

### Phase IV

To enhance the trustworthiness and relevance of the findings, and to facilitate the development of a final review narrative, we conducted 10 semistructured audio-recorded interviews with participants (managers, directors for training/development and support worker). We used a mixture of purposive, convenience and snowballing sampling to obtain the perspective of people who would reflect those with a vested interest in understanding and acting on the results. Interviews were conducted by telephone, and were guided by the content of the CMOs (see online supplementary additional file 7), audio-recorded and fully transcribed. The interviews were structured for the purposes of testing out the CMO configurations, with data confirming or disputing each mapped directly onto the CMOs and reported accordingly. All interviews were conducted by a member of the review team and lasted between 45 and 60 min.

10.1136/bmjopen-2016-011964.supp7 Supplementary additional file

## Results

Following the selection and appraisal process, a total of 76 articles were included in the study (see online supplementary additional file 8). Sixty-eight articles were located in the health and social care literature, and eight were drawn from policing and education. Eight CMO configurations were developed (box 1), which are described below and illustrated with quotes from the literature review and interview data. The CMO configurations are described separately, but the reporting reflects the interconnectedness of the configurations as a whole.

Box 1 **Eight context–mechanism–outcome configurations**

1. Making it real to the work of the support worker.
2. Paying attention to the individual.
3. Tapping into support workers' motivations.
4. Joining things up around workforce development.
5. Codesign.
6. ‘Journeying together’.
7. Taking a planned approach in workforce development.
8. Spreading the impacts of workforce development across organisations.

10.1136/bmjopen-2016-011964.supp8 Supplementary additional file

### CMO 1: making it real to the work of the support worker

We found that, where the design of interventions was intentionally focused on the role and work of the support worker, this was more likely to prompt resonance. Cognitive proximity was evident in intervention specifics or content, and judged by the extent to which the applicability of the intervention to the support worker's own work practice could be observed. Resonance with the work of the support worker was noted in reports of interventions which focused on individual older people within workers' services through, for example, the creation of biographies:^22^

Creating brief videotaped biographies of residents is an innovative way of making personal information about residents available to CNAs [Certified Nursing Assistant]. Creating videotapes of CNA/ resident caregiving interactions and using them, in conjunction with behavioral observation instruments, is an innovative way to promote CNAs' self-awareness of the person centeredness of their caregiving behaviors. (p. 697)

We found that cognitive proximity also featured in other examples, including case conference style approaches where registered professionals chose the topics and led the case presentation and discussion.^31^ Interviewees also confirmed that this helped to capture support workers' imagination and challenge their own thinking:

We're also using supervision and appraisal very much as a training tool… actually using that to really encourage discussion looking at particular case studies, so it's more like a clinical supervision. (Telephone interview: Manager)

Physical proximity involved intervention delivery in the support worker's workplace. For example, where an intervention was situated in the workplace, and designed to fit with the working pattern of the staff, being held during shift changes.^26^ This maintained

Theoretical and practical link with the daily routine of the institution. Each topic to be taken up in the training program would be closely linked to life in the institution, with the aim of fulfilling the special needs of the residents of the particular institution. (p. 591)

However, in the interview data, we also found a different perspective that suggested taking support workers out of the workplace can also be positive and provide a different learning context for participants:

Variety and change of scenery does make a difference to people's learning habits and what they learn and how they learn without a doubt, and I agree with that completely. We also have to do what works well for our organisation, within our care delivery demands as well. So it's finding that balance. (Telephone interview: Manager)

If intervention design and delivery is close to the work of the support worker (context), then this prompts resonance with individuals participating in it (mechanism), which can result in cognitive and practice changes in them (outcome). In situating interventions in the workplace, practice changes by making learning more real for the support worker. This also included paying more attention to older people. For example, visual depictions of the reality of older person's services and experiences were used in one example to encourage engagement with the intervention.^36^

### CMO 2: where the support worker is coming from

The evidence in relation to this CMO demonstrated that paying attention to the support worker's personal and role starting points (eg, background, experiences, age, challenges, existing strengths, values, abilities, and personal feelings and expectations about their work/careers) may increase their levels of engagement with the workforce development intervention. For example, in a short programme aimed at sensitising nursing assistants in a long-term care setting to ageing and the experiences of older people,^42^ the intervention focused on the self and reflection:

During the introduction, an exercise entitled “As We Grow” was used to elicit an atmosphere conducive to self-examination. This exercise required participants to write down seven of the most important things in their lives (i.e., people, animals, careers, possessions, etc.). A poem detailing the life experience of an elderly person was then read. The participants were instructed to cross off similar items on their personal list as they were identified in the poem. At the conclusion of the exercise, participants were encouraged to reflect on their feelings.

Workforce development interventions can examine support workers' personal resources (aspects about the self, linked to resilience and control^50^), and harness and build upon existing resources in a development activity:

A lot of what we're trying to do is get people to see that the skills and talents that they have outside of the service … things that can be brought to work. Maybe other residents are interested in these things, maybe they can support all different parts of life of the home and not necessarily just doing their set job, and in that way you can sort of, contributing to the sense of it being a whole home approach, having a thriving community and having lots of different kinds of varying activities going on in the service. (Telephone interview: Manager)

Paying attention to the support worker's starting points may also lead to personal outcomes for these individuals, such as confidence, empathy, self-esteem and satisfaction, which in turn can link to better interactions with older people and their families:

Is as much about the worker, as it is about the resident, and it works because they feel valued… it's reciprocation, I mean look at, it is, if you treat somebody as a human being and you listen to them and you really support them to do their best, they start to totally reciprocate with residents. (Telephone interview: Manager)

If workforce design and delivery pays attention to the individual support worker's personal starting points and expectations of the role (context), then this prompts better engagement with the intervention (mechanism). Paying attention to the individual within workforce development can promote positive personal cognitive (eg, personal efficacy) and instrumental impacts (eg, skill development) and potentially affects the organisation (eg, staff commitment) (outcome). In addition to engaging with the intervention, this approach may enhance support workers' engagement in their work.

### CMO 3: tapping into support workers' motivations

Incentivisation was noted to be a strong thread within the analysis, interpreted as efforts within the design and delivery of interventions to motivate individuals, ensure attendance and completion, and translate what is learnt into practice. We uncovered a number of ways in which support workers' engagement in workforce development was incentivised, including the use of certificates, prizes and perks, and financial/monetary investment. Incentivisation may make it more likely that participants feel they have a stake in the intervention, and feel more valued and motivated to participate, which can lead to better engagement with the intervention. Evidence suggests that lottery-style incentives (which are based on chance) on their own may not trigger sustained changes in desired workforce development outcomes. The use of financial incentives may only be effective in some service and professional contexts (eg, we found that evidence in support of financial incentives mostly related to North America and European care settings^20^ ^53^ ^54^). In thinking about workforce development incentives, there may be a need to tailor them and make them relevant to the support workers:^51^

Trained CNAs received public recognition for meeting job performance criteria … by having their names posted weekly on a CNA Honor Roll. All honor-roll CNAs listed were entered into a performance- based lottery held once each week for day and evening shifts (Reid, Parsons, & Green, 1989). For each shift, the individual winning the lottery was provided with his or her choice of incentives from a list of choices determined by each nursing home… Across nursing homes, the most frequently chosen incentives were the opportunity to leave work earlier than scheduled, extra pay, and goodie bags. (p. 453)

Outcomes from interventions involving incentivisation included increased levels of personal engagement with the intervention,^26^ and positive impacts in the quality of support workers' interaction with older people and their relatives.^52^ In one example,^26^ lottery-style incentives were found to increase personal engagement with the intervention through generating excitement about the intervention, their work and their commitment to the organisation. The incentives contributed to the development of a culture …*that supports new skills with constructive feedback and recognition* (p. 254).

If workforce development opportunities include elements of incentivisation (context), then it is likely that participants will feel recognised and rewarded (mechanism). The relationship between incentivisation and having a stake in workforce development can lead to greater emotional and practical participation and engagement with the intervention (outcomes).

### CMO 4: joining things up around workforce development

We found evidence to show that joining the organisation's strategic direction with the intervention's aims is important. Evidence underpinning this CMO included reports of organisations prioritising support workforce development to address policies,^26^ time allocation^26^ and general efforts to develop support worker roles through bespoke workforce development strategies.^32^ ^39^ There was also evidence of organisations joining up their human resource strategy with support workers' development needs. This included the development of leadership roles for senior support workers,^24^ mentorship for new staff^24^ and coaching roles, which together seek to ensure that support workers can benefit from coaching, supervision, appraisal systems and mentoring.^31^ ^32^ ^53^ In a report that described the development and pilot testing of a 6-week intervention for certified nursing assistants,^22^ the intervention was set in the context of organisational efforts to improve the quality of long-term care more broadly. This involved focusing on relationships and promoting culture change within the healthcare settings, and: …*identifying and operationalising person-centred caregiving behaviours…(p. 688)*.

Some interventions, including an advanced education programme for nursing assistants in care home settings^24^ and the development of curricula for paraprofessionals,^55^ were based on the needs of the service providers. Elsewhere, concern about the prevalence and impact of depression among older people were linked to interventions for support workers to recognise the symptoms.^40^ Here, support for staff to receive the intervention echoed the organisation's direction following concern from managers. Mutual reinforcement between the organisational goals and workforce development interventions had the potential for greater sustainability and longer lasting effects because of the types of impact achieved, for example, enhancing support workers commitment to their work,^22^ promoting better understanding of their work,^56^ ^60^ helping to develop positive attitudes towards older people,^55^ promoting more tolerance and more interest in residents' behaviours,^40^ enhancing self-reflection^32^ and leading to improvements in knowledge.^24^ ^61^

For different organisations, if interventions are developed in the context of an organisation's goals, including their human resource and quality improvement strategies (context), then this prompts mutual reinforcement between the aims of the intervention and the goals of the organisation (mechanism). This leads to more sustained and lasting impact of the intervention, reducing turnover and supporting the organisation's retention strategy (outcome).

### CMO 5: *codesign*

Engaging the right mix of people in the design of workforce development is more likely to make it meaningful, credible and relevant for the individual, and adds potential benefits for practice. It appeared from the evidence that taking a holistic approach encourages codesign and a collective approach to workforce development. Evidence showed how interventions were codesigned with a range of stakeholders. In a report of an educational programme for nursing assistants working in long-term care nursing assistants, the programme was designed by an expert panel, including physician, nurse practitioner, nursing assistant, palliative care nurse, hospice director and administrator.^27^ The authors of this article suggest that the contribution by the support workers enhanced the quality of the programme because it was made relevant to practice:

Participants suggested improvements to the content and format of the workshops, especially the provision of more concrete and practical strategies for working with families. (p. 320)

In addition to involving support workers in the design of workforce development interventions, there was evidence that highlighted the significance of involving family members:

Very often they (relatives) will have, sometimes even more of an influence we find because very often older people themselves will not like to cause trouble, will just want somebody who's kind to them, whereas actually the relatives will often come in with a slightly dispassionate view and have different expectations and standards. And so their input I think is really important. In terms of design I would say, again where I've worked in the past these things are often designed by a learning and development team of experts, but actually involving staff, managers and residents and relatives gives it a far richer input. (Telephone interview: Workforce development lead)

If the right mix of people are engaged in the design of workforce development programmes/interventions (reflecting the complexity of workforce needs and desired development) (context), this prompts codesign and a collective view about what needs to be performed (mechanism), which can lead to workforce development that is (perceived to be) more credible, meaningful and relevant for the support worker with greater potential for positive outcomes (eg, positive change) for practice (outcomes).

### CMO 6: ‘Journeying together’

Engaging with the right mix of people in the delivery of workforce development was noted to provide opportunities for learning together and promoting cohesiveness. It can lead to greater understanding of others' roles, and potential impacts on older people's perceptions of care. For example, a person-centred care programme for healthcare assistants working in dementia care used group sessions and group reflection to promote learning together.^68^ The group sessions were facilitated by registered nurses, and the pilot study enabled reciprocal learning to take place and better understanding of roles and contributions:

I thought that just being a healthcare assistant I was just a small cog in the machine. Now I feel I have an important role in the team as HCAs spend more time with patients than anyone else. (p. S62)

There was also evidence about the benefits of bringing different groups of staff together to participate in workforce development alongside support workers. Learning together also emerged from interviews. The benefits of undertaking joint workforce development for novice and more experienced support workers were highlighted:

We would not just put a course together or a classroom together of people who are all brand new to care, we like to have senior care workers who are updating or refreshing certain topics, also a mix of the two, because we feel that again it's, you have the skills and experiences being shared there, and also the people who have been working for this organisation can quickly or earlier reinforce that yes, the company's policy to do this, it's policy to do that. (Telephone Interview, Care manager)

If the right mix of people are engaged in delivering workforce development programmes/interventions (context), this can prompt learning together (mechanism), which leads to stronger cohesion across groups, greater understanding of others' roles and less duplication, and impacts on residents' perceptions of care (outcomes).

### CMO 7: *taking a planned approach in workforce development*

There was evidence to support the significance of taking a planned approach to workforce development for support workers and we noted explicit references to the use of models, theories and frameworks, and use of systematic approaches or theory to translate learning from within workforce development programmes into changes in support workers' practice. For example, in a skills enhancement training curriculum designed to improve support workers' problem-solving, communication and stress management skills,^21^ the theory of planned behaviour was linked to understanding how competency development could be transferred from an intervention to the work of the support worker. The theory of planned behaviour assumes that:

Performance of a behaviour is determined by the individual's evaluation that the behaviour will produce positive consequences. (p. 126)

In another evaluation of a training programme aimed at strengthening self-esteem and empowering staff by enhancing their understanding of factors that influence them,^28^ the intervention was underpinned by an implicit theory:

Our presumption was that one way of improving the situation for staff would be to help them develop their self-esteem and feel empowered though a training programme. This programme focused on helping participants to understand factors in the work situation that influence them and on empowering them. (p. 835)

For different organisations, if workforce development draws on theory (explicit and implicit) or there is evidence of a planned approach (context), this prompts the adoption of a systematic process in its design and delivery (mechanism), which leads to greater potential to demonstrate impact, and learn about workforce development effectiveness (outcome). In this CMO, theory could be associated with taking a more systematic approach to workforce development, which meant that the achievement of learning outcomes was made more obvious within programmes, and a key requirement for wider programme evaluation and process learning about improving workforce development.

### CMO 8: spreading the impacts of workforce development across organisations

Workforce development programmes/interventions that are comprehensive (ie, multilevelled and with more than one component) have the potential to prompt attention being paid to the way in which interventions/activities reinforce one another. Efforts to demonstrate a comprehensive approach to workforce development were evident in linking elements to the wider context of the organisation. This was reinforced in interview data where we found reference to longer lasting impacts of workforce development if focused across the organisation:

We find that anything to really have a lasting impact it's got to be something that's a whole home approach, so if we're doing something with the support workers we also need to be working separately with the managers, with the activity leads, and we need to be doing that over a long period of time, because otherwise it's a limit to how much it becomes an everyday way of working…they need to see that other people want to do it, that their manager is talking about it in staff meetings, celebrating it when they're doing something that's been a learning from the course. And that only happens if… joined up. (Telephone Interview: Manager)

Data were included from practice development programmes,^71^ which work at multiple levels (individual, team and organisation), so that there is potential to create impact at an organisational level, which could last longer than one-off interventions aimed at the individual support worker. There were some (albeit limited) examples of workforce development approaches that were more comprehensive, for example, by not only incorporating the individual support worker perspective but also addressing their role (and impact) within groups, teams or the organisation as a whole to show how interventions can reinforce one another. This finding was prominent in articles that featured, alongside the reporting of the intervention, evidence about innovation leadership, mentoring, supervision and team functioning.^26^ ^32^ ^52–54^ ^62^ ^68^ ^71^ Some support worker development was nested within the development of other workers and organisations as a whole, with the implication that development at one level is inherently linked to development at other levels.

For different organisations, if workforce development interventions are comprehensive, in that they are multilayered (focusing on individuals, groups and organisations) and reflect broader developments relevant to the support workforce (context), then this prompts attention to the way in which components of interventions reinforce one another (mechanism), increasing the potential for impacts to embed and spread across organisations (outcome).

## Discussion

The review findings have resulted in the development of a programme theory, grounded in evidence from the literature and stakeholder perspectives, about how workforce development works in improving outcomes for support workers, their employing organisations and older people's services. The results provide a plausible, credible and evidence informed account of what works, how, why and in what circumstances. While current guidance calls for flexible local learning and development opportunities for the support workforce,^72^ in reality, this may not always take priority. For different support workers, operating across a range of diverse settings, and where lack of time or priority for their development may be problematic, we argue that the findings from this review can help support and guide managers and services to develop the workforce in older people's services. The inclusion of material and examples drawn from the reality of practice and integrating learning within the expectations and boundaries of support workers' role is important.^10^ Theories of adult learning already emphasise the importance of the self in shaping how we learn.^73–76^ Our findings show that if workforce development interventions are constructed to build on the life skills and experiences that individuals bring to their role, this is more likely to enable role development and career progression (if this is desired by the individual) for the support worker and their organisation.^10^ We found that, if the opportunity exists, it is useful to incorporate strategies and techniques that might incentivise and motivate individual engagement in the intervention/activity.^10^ In self-determination theory, intrinsic and external factors can influence motivation. Although there has been some debate about the potential for extrinsic factors, such as the reward-based incentives uncovered in this review, a recent meta-analysis indicates that both are important.^77^ Incentives may be effective in influencing participation in workforce development, and intrinsic factors may be crucial in ensuring the quality of participation in the process.^10^

We recognise that workforce development programmes operate in a given context, where that context or set of conditions represents a mix of social, cultural and material factors. Our review findings suggest the importance of taking a systematic approach to the design of workforce development, one which is aligned with organisational strategy around, for example, priorities such as service quality and integration across health and social care.^10^ Our findings resonate with broader ideas about the benefits of coproduction and imply that workforce development can be designed and delivered in a coproductive approach involving relevant stakeholders, including the support workers themselves and those that they work with, from the beginning of the process. Different stakeholders bring varying priorities and expectations to the design process in workforce development, and may draw on and contribute different knowledge bases which, cumulatively, enrich the learning process and environment.^78^ Involving lay stakeholders can be important and there are different theoretical explanations of their impact on workforce development.^10^

Finally, workforce development can often be considered as a complex programme that is transformative of people and organisations; therefore, it should not be ad hoc and fragmented. We found that the design and delivery of workforce development intervention for the support workforce can often be approached in a theory-driven and systematic way, including reference to, and inclusion of, relevant theory/ies, and frameworks and the learning methods/approaches/tools used linked to those underpinning heuristics.^10^ Workforce development also needs to be framed in the context of the whole system, which includes individuals, teams and the organisation in its wider context. Key features of complexity theory that are relevant to the implementation of workforce development interventions include understanding behaviour of the whole (system) rather than its constituent parts.^10^

### Implications for practice

From the review, it is clear that a number of points warrant attention in the context of current health and social care policy and practice.

Where the challenge is about how to design and deliver workforce development:

- It is important to consider the broader organisational strategy and goals and consider how the development need or gap aligns with the needs and strategy of older people's services, workforce development plans, and the adaptation of health and social care policies/procedures for local needs and ways of working.
- Consider the specific requirements of the workforce development challenge in the context of improving the service for older people—including where the focus for change comes from (eg, older person, family, carers or support workers) and the development needs, which may be clinical, technical, behavioural, cultural, individual, team or organisational.

When the challenge is to promote individual engagement with workforce development:

- Consider personal factors about the support worker—including their personal background, career aspirations, their existing strengths, including life skills, development needs, values and experience.
- Workforce development interventions need to be organised to reflect the realities of the support worker role in different circumstances.

### Strengths and limitations of the study

We consider that using the realist approach for this review was a key strength. The philosophical underpinnings of realist synthesis focus on theoretical depth, breadth and transferability, rather than a quantitative account of the contribution of each CMO configuration within the programme theory. A second strength of this study was the embedded approach to stakeholder engagement. The realist viewpoint accepts that social programmes are underpinned by a variety of resources, opportunities and barriers for different groups of stakeholders. In this review, stakeholders were involved in a process of prioritising, and refining the theory areas and making additions. Additionally, we engaged with stakeholders throughout the synthesis process to ensure we maximised relevance. An added strength was the inclusion of other fields (education and policing) in the search to seek data about similar mechanisms of action.

We hope that future application of realist methodology can draw on our account of the approach to this review, using the tools and processes described in this article. Our tools include a living document to log decisions and reflections, and a set of constructs within the data extraction form to guide decision-making. Soft systems methodology guided our understanding of factors which we found can influence the success or otherwise of workforce development at a system level. Our engagement processes included additional support for decision-making from the wider team in our regular monthly meetings, and active engagement and communication with stakeholders and Patient and Public Involvement (PPI) representatives through, for example, workshops and group work. Transparent reporting of the analysis and synthesis process in realist work is challenging. We used abductive and retroductive reasoning to illuminate what was happening within and across the CMOs.

From a methodological perspective, we acknowledge the challenges of conducting a review about topics entwined within complex social situations. Our results were limited by the nature of the evidence base. We found that reports of studies evaluating workforce development interventions tended to lack detail about the interventions themselves. Further they lacked specificity about the perceived and actual intended impacts from the workforce development initiatives being implemented and/or evaluated. This challenged our work to make inferences regarding the CMO configurations and development of programme theory. However, the inclusion of stakeholder engagement and interview data in phase IV complemented and greatly informed the process.

### Recommendations for future research

Our recommendations for future research relate to the process of describing and evaluating workforce development interventions. The synthesis demonstrated generally poor reporting of workforce development interventions; therefore, in future research, we suggest that the recommendations proposed in this synthesis could be used to describe the nature of the intended workforce development. Authors need to provide clear and detailed descriptions of the component(s) of the intervention. Adopting our recommendations would help to ensure that the theory of change for the workforce development intervention is clearly reported.

## Conclusion

In conclusion, we believe that the programme theory that has emerged from this review has the potential to improve workforce development for support workers, and subsequently, older people's experience of care, through shedding light on what works, for whom, how and under which circumstances. The programme theory highlights a number of starting points to increase the potential of sustained impacts for support workers, older people and service providers. Intervention components and activities need to be relevant to support workers and their work, joined up and inclusive of examples/experiences from the reality of practice. Workforce development can incorporate learning alongside peers or others, with space for sharing, communicating and working on challenges together. Incentives may offer meaningful intrinsic and extrinsic rewards for engaging with development opportunities and recognising achievements. Codesigning and codelivering development opportunities recognises people's different perspectives and provides an opportunity to build a platform for shared learning. In the context of national debates about the future of support worker roles, and ongoing concerns about the quality of older people's care services, this review provides a timely contribution in terms of a set of robust principles for developing the skills and knowledge of support workers.
