# Supplementary material for: Improving skills and care standards in the support workforce for older people: a realist synthesis of workforce development interventions
Source: BMJ Open. 2016 Aug 25;6(8):e011964. doi: 10.1136/bmjopen-2016-011964 (PMC5013423; doi:10.1136/bmjopen-2016-011964)
Supplement: Supplementary additional file [file bmjopen-2016-011964supp1.pdf]

Additional file 1: Long list of issues in theory areas

**1. Workforce development will be most effective when it is aligned with organisational and other career development frameworks, and opportunities and benefits for individuals, teams and organisations may have greater visibility and meaning**	**Fleming et al (2006): Commissioning brief, (2012): Cavendish (2013)**
Theory area questions:<br><br>• *What impact does career development have on outcomes for the workforce?*<br><br>• *How do the ways in which support worker roles and personal development are met through workforce development impact on outcomes?*<br><br>• *How do organisational and work factors mediate on the outcomes for workforce development, services, older people and their care providers?*<br><br>• *How do the means in which workforce development interventions are streamlined/connected impact on outcomes for the workforce, service, and older people and their care providers?*<br><br>• *What is the impact of support worker roles being promoted as a positive career option?*	
**2. Effective workforce development is designed using flexible, interactive, experiential approaches, and implemented and evaluated in close proximity to the work of the older people's support workforce and practice. *Programmes or interventions that are not grounded in the reality of daily work completed by the older people's support workforce, nor delivered within in the workplace, are less likely to be effective***	**Smith et al (2005): DoH, (2008): Commission on dignity in care for older people (2012)**
Theory area questions:<br><br>• *How does the location of workforce development impact on outcomes for the workforce, delivery of*	

services, and older people and their care providers?<br><br>• *How does the flexibility in delivery of workforce development interventions/programmes impact on outcomes?*<br><br>• *How do educational models and styles impact on the outcomes for workforce development?*<br><br>• *How do the ways in which support workers are involved in setting the workforce development agenda impact on the development of support worker roles?*	
**3. Effectiveness can be mediated by the personal characteristics of members of the older people's support workforce, practice development aspects of human and social geography; characteristics of the organisations in which the support workforce are operating workforce and service policy; and public experiences and expectations**	**Prochaska & DiClemente, (1982): Easterby-Smith, (1997): McCormack et al (2007):Dewing(2008)**
Theory area questions:<br><br>• *What impact do the people involved in the design and delivery of workforce development have on the outcomes for the workforce, service and older people and their care providers?*<br><br>• *How does workforce development enhance the public perception of support workers' roles?*<br><br>• *How do the role of personal and other characteristics of the workforce influence workforce development outcomes?*<br><br>• *How do leadership styles impact on the outcomes for workforce development?*<br><br>• *What is the impact of policy e.g. health and social care on the outcomes of workforce development for the workforce, services, older people and their care providers?*	

• *How do strategies for commissioning, validating, accrediting, and quality assurance impact on outcomes for workforce development, services, older people and their care providers?*<br><br>• *What is the impact of multi-cultural issues on the outcomes for workforce development in this context?*<br><br>• *What is the impact of gender issues on the outcomes for workforce development in this context?*	
**4. The effectiveness of workforce development interventions, and programmes for the support workforce for older people's services can span outcomes for: the workforce the delivery and quality of services through organisations and for older people and their families/ care providers. Interventions/programmes are most effective where positive impacts from workforce development can be identified in all the above areas.** ***Where these impacts do not meet the expectations of older people or health and social care organisations, then positive, individual changes from workforce development programmes/interventions might be evident and /or achieved, but not be sustained***	**Nancarrow et al (2010): Commission on dignity in care for older people, (2012): Commissioning brief,(2012): Cavendish, (2013):**
Theory area questions:<br><br>• How do workforce development interventions balance core and specific skills for the support workforce for older people's services?<br><br>• How do programmes instil and encourage confidence in the support workforce to contribute to quality improvement?<br><br>• How do workforce development interventions for support staff develop the concept of relationships in older people's services?	

• How do workforce development interventions impact on outcomes for the workforce (i.e. motivation, job satisfaction, self-esteem and confidence)?<br><br>• How does workforce development meet the current and anticipated knowledge, skills, and attitudes required of the support workforce?	
