# Supplementary material for: Improving skills and care standards in the support workforce for older people: a realist synthesis of workforce development interventions
Source: BMJ Open. 2016 Aug 25;6(8):e011964. doi: 10.1136/bmjopen-2016-011964 (PMC5013423; doi:10.1136/bmjopen-2016-011964)
Supplement: Supplementary additional file [file bmjopen-2016-011964supp3.pdf]

"This work is supported by the National Institute Health Research (NIHR) HS&DR Programme Grant no. 12/129/32. This report presents independent research funded by the National Institute for Health Research (NIHR). The views and opinions expressed by authors in this publication are those of the authors and do not necessarily reflect those of the NHS, the NIHR, NETSCC, the HS&DR programme or the Department of Health"

Older People's Services and Workforce Interventions: a Synthesis of Evidence

"Relevant and good enough" flow chart

1. Does the evidence link to one or more of the study's programme theories?

Does it match?

("Fidelity")

____
____
____
____
____
____
____
____
____
____
____

→ **1. Evidence around the impacts of career frameworks, policy, organizational developmental frameworks Reported outcomes for individuals, teams or organisations.**

→ **2. Evidence of impacts from the design & delivery of workforce development interventions.**

→ **3. Reported impacts from support workers' personal characteristics, workforce and service policy, public experiences and expectations.**

→ **4. Outcomes for older people and their families (around the quality of service, workforce,organisations.**

↓

In your judgement:

2. Does the evidence provide valuable information? ("nuggets")

____
____
____
____
____
____
____
____
____
____

→

3. Can the evidence be relied upon?

("trustworthiness")

____
____
____
____
____
____
____
____
____
____

→

4. Is the evidence what is needed? Does it contribute to the study?

("relevance")

____
____
____
____
____
____
____
____
____
