# Supplementary material for: Improving skills and care standards in the support workforce for older people: a realist synthesis of workforce development interventions
Source: BMJ Open. 2016 Aug 25;6(8):e011964. doi: 10.1136/bmjopen-2016-011964 (PMC5013423; doi:10.1136/bmjopen-2016-011964)
Supplement: Supplementary additional file [file bmjopen-2016-011964supp4.pdf]

Additional file 4 –Evidence table example

Evidence source	Theory area 1: Fit with career, development, policy, strategy and structures
1. Arblaster et al (2004)	The report authors suggest that career development for healthcare assistants is best promoted through using a phased approach to Continuing Professional Development (CPD) and progression, and linking national strategy to frame design (Essence of Care, in this case);<br><br>“the training and development pathway incorporates three phases of continuing professional development: the Essence of Care programme, and NVQ Care levels 2 and 3. These enable HCSWs to develop skills and achieve recognition of these skills, with the opportunity to progress to nurse training. The pathway was presented as a progressive flow chart that indicated the development of HCSWs from induction through to the exit point for nurse training” (p34).<br><br>The report’s authors suggest that the academic gap is addressed through linking with a study skills module as integral part of developmental pathway for Health-Care Support Workers (HCSWs), based on collaboration with Higher Education Institutes (HEIs), and discussions with student nurses who had completed the National Vocational Qualification (NVQ) route. The report implies the benefits of a structured approach to progression in an NHS Trust (United Kingdom (UK) context), and use of manager nomination model for progression to the next level;<br><br>“implementing a structured approach to the training and development of HCSWs ensures gradual development in both theory and practice. It also enables HCSWs to plan their professional development and identify a career pathway” (p37).<br><br>Due to the organization’s policy of employing large numbers of HCSWs, the report noted discrepancy in training consistency (NVQ versus “on the job”).<br><br>The report’s authors place emphasis on linking with partners to deliver training programmes (using practice facilitators, specialist nurses, external agencies), and highlights the importance of organisational commitment to develop competency and consistency in care for healthcare support workers.<br><br>This paper reports how support for HCSWs is often reliant on the support they receive from their peers in practice. A lack of qualified nurses as assessors because of work demands led to healthcare support workers becoming assessors themselves;<br><br>“It was apparent from discussion with HCSWs that much of their support in practice came from successful NVQ Care candidates who had experienced the process first hand. This suggested that there was the potential for HCSWs to train as NVQ Care assessors” (p35).<br><br>Managerial support was noted as significant to reduce; “the potential risks associated with devolving responsibility for assessment of competency in the

	skills required to deliver fundamental care from registered nurses to unqualified staff" (p35).
2. Aubry et al (2012)	The paper authors (based in Canada) highlight the challenges for the development of support workers entering long-term care services, where the focus on innovation is driven by policy and academia.<br><br>Mismatch between training and reality; "'It's impossible; trainers don't see the reality of things in long term care facilities…' We were told that we had 45 minutes per resident but we actually have no more than 15 minutes. In short, they saw the gap between the value of care, which takes time, and the value of work efficiency" (p6).<br><br>The data showed that experienced nursing assistants played an essential role in the transmission of productivity requirements to new recruits. Early stages of the recruits' integration into the ward behaviours were spent on the orientation or training phase. This was a short period of time (two to five days) during which the new recruit worked under the watchful eye of the referent, experienced nursing assistant. A staff member in charge of new recruit activity shared this;<br><br>"I'm in charge of the orientation of new recruits […]. We look at recruits working; we see how they are when they start; we see what happens. It takes a certain pace, it's Go! A toileting, go! Another... And we help them to keep the pace because they don't learn that during their training" (p7).<br><br>Linking systemic change management and multidisciplinary approaches are believed to be essential to drive innovation in Long-Term Care Organisations (LTCOs) whereby;<br><br>"implementing change in LTCOs remains a complex task that is often based on intuition and anecdotes and characterized by very uncertain outcomes" (p2).<br><br>The authors used the strategic analysis approach to understand the relationship between the development of nursing assistants in long-term care services and the organisational context, and how this relationship impacted on developing change management strategies;<br><br>"the strategic analysis framework explains that collective action is defined not only by the prescribed work, namely post-training 'good' practice, but also by the workers' ability to build innovative practices to meet work requirements. Although Canadian (older people's care) managers control the entire work process, the aides agree on their daily tasks and the distribution of these tasks. Crozier's framework makes it possible to define the means of understanding the collective structure of the Nursing Assistants' work during the many change endeavours facing LTCOs, as described below in the study findings" (p3).<br><br>The study authors suggest that challenges and constraints can lead to homogeneity amongst Nursing Assistants (NAs), showing how they develop

	(informal) strategies, and how these are shared with new staff. The study finds that using informal practices and team working can facilitate change;<br><br>"overlooking the potential input of nursing assistants in the development of an innovative care program or else soliciting the input of an individual nursing assistant, rather than that of the 'team', can foster resistance to change" (p9).
3. Braun et al (2005)	The study authors (based around home/ community health-care direct care workers with older people in the United States (US) in non-Medicare ageing network) suggested an identified gap in nonclinical training/ education;<br><br>"our goals were to provide a foundation for nonclinical direct care workers by imparting a basic knowledge of aging, improving assessment and reporting skills, increasing empathy for older adults, teaching strategies to manage stress, and validating the importance of direct care workers" (p119).<br><br>The study authors report how disjointed approaches can lead to lack of meaning for direct care workers;<br><br>"direct care workers in this study had relatively few years of education and little knowledge of basic aging processes, differential effects of aging and disease, and good self-care principles. Many conceptualized their jobs as custodial, rather than therapeutic, and did not see the value of their work" (p122).<br><br>The study authors identify how caring for *themselves* can impact on the care that direct care workers provide for older people (p119). In this study, the degree of control direct care workers had on their development and practice influenced the success of the intervention;<br><br>"another probable reason for success is that we focused on improving basic knowledge and attitudes that were under the control of the direct care worker, rather than on teaching clinical behaviours that may require concurrent organisational or systems change" (p123).
7. Coogle et al (2007)	The study authors reports on the significant linkage through multiagency collaboration to the development of the curriculum for care staff;<br><br>"a joint project advisory group was formed that included representatives from the Virginia Association for Home Care, the Virginia Association for Professional Nursing Assistants, other home care networks, and a large provider of services to clients under Virginia's (older people) and Disabled Medicaid. This initial collaboration of pertinent stakeholders was crucial to the development of the training program (Lewis, Rachel, Coogle, & Parham, 2004). Bringing together the strengths and interests of multiple parties in academia, provider agencies, and professional associations garnered state-wide support for the effort and ensured that concerns were addressed" (p112).

	The study findings helped to identify that, since the training was designed to encourage a more career-focused orientation, enhance professionalism, increase self-efficacy, improve self-worth, and maximize intrinsic motivational processes, it is likely that the improved application of training content resulted in a deepened commitment to care work as well which was;<br><br>"'grounded in an intention to encourage a more career-focused orientation' And 'enhance professionalism by enabling support workers to better handle challenging situations, aggressive behaviour, and work-related stress'" (p115-116).
8. Cowan et al (2004)	The study authors link the development of effective learning strategies with eventual nurse registration, practice and post-registration continuing education, to facilitate access to lifelong learning for support workers (SWs) employed in care homes for older people in the UK (linked to European Commission directive on lifelong learning).
12. Hegeman (2003)	The study author reports how a not for profit organization was enthusiastically supportive of the project in long-term care setting in the US. The author linked the peer mentoring programme to the improved culture within the organisation as it had the potential to improve the retention of staff where turnover of staff was high, which also ultimately reduced costs for the organization;<br><br>"peer mentoring is also-in itself-a way to create a culture of caring among aides within a long-term care facility. When carefully-selected, experienced CNAs who embody the caring values of their facility become successful peer mentors, there are clear potential organisational changes" (p186).<br><br>Certified Nursing Assistants (CNAs) who demonstrate a culture of caring were recognized as such, thus increasing their own satisfaction (and supporting retention);<br><br>"the behaviours and attitudes the new CNA is exposed to, making it more likely they will be the traits which are learned and emulated" (p187).
29. Hockley (2014)	This paper was a report of how to develop strategies to develop quality end of life care in nursing homes. Time constraints, low morale and need for culture of learning were identified as work factors during the action research.<br><br>Experience-based learning was considered to be a means of drawing out the ways in which people learn – including learning through didactic instruction, experiential exercises and support to enhance critical thinking (p125).<br><br>The study highlighted the importance of the provision of support and learning for care assistants;<br><br>"Care assistants play an important part in end of life care because they have often cared for the resident(s) for many months, even years. They can feel excluded when nurses "take over the care" when a resident is dying (Hockley et al, 2005)" (p126).

14. Morgan & Conrad (2008)	The study authors found that the ratings of perceived career rewards improved for participants who completed a specific programme in North Carolina nursing homes. The authors imply that the programme strengthened view of care work as career opportunity, not "just a job".
15. Nelson & Wild (2009)	The study authors found that union membership and conditions of employment were perceived as being important by care staff participants (UK based paper). Participants;<br><br>"recognized a current lack of professional status, regulation, and accountability associated with care work: 'Should be able to be part of RCN (Royal College of Nursing)—we are a missed and forgotten group'; 'Feels like a back door profession—that anyone can enter. Should be regulated like nurses and be able to be struck off'"(p202).<br><br>The study authors identified issues around care staff development, accountability and scope of practice;<br><br>"Care staff seemed divided in their views on how universal such awareness was. Some new role care staff showed awareness of the links between responsibility, accountability and liability, e.g.' More questioning now, more assertive and aware, more realisation that it is our head that rolls', but this was not universal: 'Don't think people realise the responsibility.'; 'Think improvements should be made re insurance'" (p202).<br><br>Issues were identified in the paper around relationships and ways of working –implication is made that care assistant development roles require time to establish the carers' new roles but also for the in-reach team to overcome various issues with community nurses, including ways of working to avoid duplication of activities.
30. Nilsson et al (2014)	This paper's authors reported an e-assessment of prior learning: a pilot study of interactive assessment of staff with no formal education that are working in Swedish (older people's) care.<br><br>A third of all staff working in Swedish older people's care lack formal competence for their jobs (p2). Staff are often working temporarily, putting extra demands on organizations and permanent staff. All staff working in older people's care have to show basic professional skills for the tasks involved (p2).<br><br>Upper secondary school care programme curriculum seen as benchmark for national standards. Assessing informal/ non-formal learning seen as way of improving lifelong learning (p2).
16. Noelker et al (2006)	In this study of NAs in care settings, in Ohio, US , the link is made between supervision and turnover;<br><br>"associated high turnover with poor supervision by licensed nurses who typically receive little or no training in management skills such as leadership, motivation, and team building" (Eaton, 2001; p308).

	The study authors highlight the significance of managers showing respect towards NAs.<br><br>Assumptions are made about the influence of the characteristics of the workplace;<br><br>"it is expected that personal and job-related stressors will be more widely reported by NAs working in for-profit facilities compared to non-profit facilities based on research indicating that staffing practices and working conditions in for-profit facilities place greater demands on nursing staff (Anderson, Issel, & McDaniel, 1997)" (p309).<br><br>The paper authors allude to the general lack of training that was available for nursing assistants;<br><br>"the required minimum 75 hours of entry-level training is widely regarded as inadequate to train workers properly (Eaton, 2001; Stone & Weiner, 2001)" (p310).
17. Noelker et al (2009)	The study authors identified a lack of training for supervisors in care settings, Ohio, US;<br><br>"information collected from 139 nurse supervisors of these workers as part of the larger study showed 47% (63) had no formal training in supervisory practices and, of those who did, only 26% (19) felt this training made them well prepared to work as a supervisor. The continuing education these supervisors received also was reported as lacking. More than half the supervisors said their continuing education either did not cover or was not helpful in improving their abilities to do the following: demonstrate good leadership, handle racial issues in the workplace, motivate staff, manage insubordination, and deal with difficult workers" (p94).<br><br>Organisational factors were considered to be influential;<br><br>"the positive effects of peer mentoring at hire and having job orientation and continuing education programs that workers view as adequate for their needs suggest these programs should be given more careful attention in efforts to improve satisfaction with supervision" (p98).
20. Petterson et al (2006)	The study, set in nursing homes in Sweden, includes ,together with reporting of findings, the use of stress theory to plan interventions, hypothesizing that;<br><br>"strengthening staff resources of job control, work support, and learning (Karasek & Theorell, 1990), as well as attaining new coping skills, is health promoting" (p354).<br><br>"Applied to worksite health promotion, empowerment has much in common with the Karasek stress model, emphasizing that work related stress is reduced by increasing employees' control over work. Job control is defined as learning authority (access to competence and new learning) and authority

	over decisions (work influence and participation). By increased job control and support, the self-confidence of employees is enhanced, stimulating their engagement and activity" (p356).<br><br>"The project has inspired new efforts on professional authority and career development for auxiliary and assistant nurses. Advancement opportunities have been created for these nurses by a recently permanent stepwise career ladder, which serves as a model for (older) care organizations in other municipalities in Sweden and has attained international attention" (p.367).
23. Stevens & Hochhalter (2006)	Staff development programmes (in nursing homes, in the US) should;<br><br>"recognize the complex interplay between the personal competencies of residents and staff and their environments" (p249).<br><br>The authors refer to staff training programmes that can change; "residents' environments to better match residents' competencies" (p249).<br><br>The Informed Teams model is discussed whereby; "active organisational support is a prerequisite" (p253).
24. Stevens-Roseman & Leung (2004)	The study authors focused on paraprofessional training for staff working with older people in the US, and highlighted the significance of the organisational/ manager role of reinforcement;<br><br>"Reinforcement is relevant in two ways: (1) reinforcement of learning promotes more learning, and (2) training processes modelled the reinforcement of strengths. By reinforcing paraprofessionals' strengths and worth, trainers convey the impact and the process of building on strengths. This skill can then be applied by trainees in recognizing the worth of (older people) during the course of their work" (p86).
26. Tisher et al (2009)	The study, based in an older people's care setting in Australia, showed evidence to suggest that organisations need to support training with shift in culture, supporting staff to manage workloads, emotional burden, and allowing time for staff to share experiences. In particular, the authors refer to an organisational role to support staff to develop more cooperative relationships with families.
28. White & Cadiz (2013)	The study of direct care workers in Assisted Living (AL) in Oregon found that a focus on the environment is significant to success– for example, investment in supervisors, organizational policy changes made to support trainees (e.g. tuition advancement);<br><br>"In addition to the content targeting AL workers, the results of this study offer additional support for a work-based learning design for frontline workers. The emphasis on one-on-one and small group learning provides a structure for engaging and supporting all levels of staff, both as trainers and learners. Although a modular teaching approach has been used successfully in many LTC and other health settings (e.g., see Stone et al., 2002), it is particularly important for AL settings with their limited training resources and

	a workforce often characterized by low literacy and non-native English speakers" (p296). NB: LTC = Long-term Care
42. Clarke et al (2003)	Support workers are included in this paper which describes the findings of a developmental study undertaken over a 6-month period to investigate the introduction of a biographical approach to care on a unit in a NHS hospital;<br><br>"the focus of the study was on the experiences and views of older people (and, wherever possible, their families) and practitioners working in the field. Practitioners were central to the research process and were involved throughout the planning, delivery and dissemination of the study. Staff included both Registered Nurses and support/ care workers who volunteered to participate in the study. From the outset, it was agreed that support workers would work with the older person and their families in undertaking biographical work, as they spent the most direct time with patients' care-related activities" (p700).
45. McLellan et al (2005)	This report's authors describe how a new inter-professional Primary Care Practice Team defined and implemented a new appraisal system drawing on the principles of 360 degree appraisal;<br><br>"We recognized that by tailoring the 360 degree appraisal method to MMP (name of Primary Care practice) needs we could aim to:<br><br>▪ support and reinforce positive and responsible inter-professional team working<br>▪ take into account the concerns and requirements of both the team members and the service organization<br>▪ embrace the appraisal requirements of different professional groups and partner organizations<br>▪ accommodate all members of the MMP team within one appraisal system<br>▪ introduce a sustainable system which spread the workload associated with appraisal and did not overburden individual team members<br><br>The proposed 360 degree appraisal process would be complementary to the ongoing systems for management and professional development in place at MMP. These included short monthly one-to-one meetings between the service development manager and each team member to discuss workload, performance and development, and also team discussions about roles, responsibilities, values, team policies and service developments" (p140).
46. Parry & Vass (1997)	This paper's authors discuss some of the issues relevant to the role and training of physiotherapy assistants. It describes the processes of role definition, assessment and training of one particular assistant, developed in the context of a larger research study. Evidence about assuming capability of assistants, and importance of supervision, are reflected in the interviews with physiotherapists;

	“A number seemed to imply a belief that the potential of assistants is underestimated, as illustrated by this comment: ‘We shouldn’t underestimate what they should be capable of, just because [they’ve] not had lots of academic training. In neurology it’s handling that’s important.’ Important qualities in assistants mentioned by some were aptitude and abilities, and by others, experience. Two interviewees remarked that level of experience is not necessarily linked to aptitude. Interest, enthusiasm, and ability to learn were also mentioned. Understanding of boundaries was an important theme: ‘Initiative but with the right perspective... [They need] awareness of the limitations of the role.’<br><br>Having correct expectations and knowing your staff was mentioned as important by several, as was the need to give assistants responsibility when treating patients, and a role within the team.<br><br>‘Level of supervision depends on competence and experience ... It’s a professional judgement (that’s true of supervising juniors too!)’ ‘They need to have a feeling of positive contribution- involvement in decision making.’” (p36).
48. Vail et al (2011)	Evidence provided relating to theory area 1 about the Health Care Assistant (HCA) role in general practice. The paper authors consider how progression, acknowledgement of the role, and personal characteristics sometimes influence development.<br><br>The report emphasises the importance of recognition and the environment for role development. The data revealed attitudes towards being a HCA, and in particular, the younger participants spoke more negatively about lack of opportunities to progress;<br><br>“Although the three youngest HCAs were positive about their role and claimed to enjoy it, they spoke more often about negative aspects of the job, such as low pay and the lack of opportunities to progress.<br><br>‘I think we should be on more money we’re at risk from needle stick injuries and infections and all the rest of it. And we’re on fourteen and a half thousand [pounds].I don’t understand how they got to that figure. I think we’re undervalued for the skills that we’ve got’ (HCA5, age 24).<br><br>‘I do get frustrated because there’s only a certain amount I can do without my general nursing qualification. I’m actually hoping to go to University. I’d like to learn more and do more’ (HCA14).<br><br>‘[T]here’s no way you can advance up the [salary] bands so you’ll actually earn more money. So then you think, Where’s the incentive to do any training?, I could just do what I was doing last year, which was not very much, and stay on the same money’ (HCA12, age 27)” (p34).<br><br>Concerns were expressed by some participants about the limitations of the HCA role;

	"'[I]t's being not able to go further and get that extra knowledge that you want, you know, when you get so far and you just y you're hungry for it and you can't get it' (HCA3).<br><br>A lack of awareness and understanding within the GP team about the HCA role was another source of frustration. Two of the interviewees, both of whom had come from practices in which the role was new, reported that their colleagues were initially unaware of what tasks could be delegated to them. 'Here was a little bit frustrating for me when I first started because they were y they were unaware of what I was and what I could do y because they'd never had a Health care assistant before' (HCA11)" (p35).<br><br>The paper sheds some light on the perceived status of HCA role;<br><br>"Over half of the HCAs commented on their role being of minor importance within the GP team. The interviewees often referred to their tasks as 'menial', 'silly', and 'mundane', and implicitly or explicitly compared them to the 'more important' responsibilities of the nurses. Many felt that the main purpose of their role was to provide help for the nurses.<br><br>'I think I take the pressure off the nurses because I can do little jobs for them, which means that they can move on and do other things that are more important' (HCA2).<br><br>'Obviously, I'm not allowed to give injections and do smears and that, but just silly little things like doing a leg dressing or stitch removal' (HCA1)" (p35-36).<br><br>"There appears to be a need for greater awareness of the HCA role among practice team members in order that the potential benefits of the role can be fully realised. Practices, therefore, need to ensure that their wider workforce is fully prepared when taking on an HCA for the first time. HCAs in this study appreciated their colleagues' support and encouragement on both a day-to-day basis and also in relation to role development. This indicates the importance of providing protected time and resources for mentorship, for ensuring that HCAs feel valued in their role, and for supporting their career development. Furthermore, this highlights the significance of effective inter-professional working for ensuring that HCAs are satisfied within their role" (p39).
52. Ryan et al (2004)	The authors of this paper, based on interview data gathered from a group of community-based dementia care workers, seeks to identify their sources of job satisfaction and reward.<br><br>The authors provide ideas around the organization which were considered to promote job satisfaction for support workers. These ideas could be considered as important for individual and team development. Factors which supported support workers through promoting job satisfaction were;<br><br>"open channels of communication between the support workers and more senior colleagues, which facilitated exchange of information, advice and ideas. Support workers particularly valued opportunities to explore 'difficult'

	situations with other team members. A 'free and easy' atmosphere was described, enabling team members to talk openly and honestly: You can ask or talk about anything without feeling embarrassed. I think if we are ever struggling with a problem you always feel that you can ask someone who might have had that bit more experience, ask for advice here and there, which direction you should take" (p113).<br><br>"weekly team meetings were seen as essential in helping to engender cohesion and provided the forum for much of the above discussion. However, one support worker noted that things could be improved if attendance at team meetings was made mandatory. It was also suggested that the office was perhaps not the best venue for open discussion" (p114).<br><br>"support workers stressed that the ready availability of more senior colleagues was pivotal to them feeling well supported, especially at difficult times. This fostered a feeling of security amongst the staff team as well as contributing to a thorough assessment of risk (for both staff and users of the service), thereby helping to reduce potentially difficult incidents from arising. While formal supervision occurred on a monthly basis, it was the availability of senior colleagues at any time that was most valued:<br><br>'You do have problems at times, but she [the service manager] is always there. I always speak to her … I say to her, 'I bet you think I'm a pain don't you?' [laughs], but she says, 'Oh no.' It is best to clear it, especially if there are potential problems, clear it, discuss it'" (p114).<br><br>"Thirdly, a strong sense of purpose and clear understanding of the aims of the service amongst the team, together with being valued, were clearly central to the success of the service. It was not just immediate managers who valued the contribution of the support workers since senior personnel within the National Health Service trust were also perceived to value the service and support the workers' contribution to it:<br><br>'If you've got a service which is promoting good care and support for the people they are looking after, then that should go too for the people that work in the service. Everybody [within the team] feels valued and that their work is worthwhile, and that can only help'" (p114).<br><br>"These data exemplify the significance of being valued by fellow colleagues and senior managers, of feeling protected from potential overwork and emotional harm. This was essential because the support workers were relatively autonomous in their day-to-day work, which was another major factor contributing to their feelings of job satisfaction" (p114).
49. Bailey et al (2013)	This paper provides insight into the emotional labour of the HCA role and how HCA skills to detach themselves from the emotional burden of their work can be a positive component of engagement.<br><br>Ideas are offered for teaching/ supervision. The paper explores the concept of detachment in a positive light, offering case studies to illustrate points;

	"Through these cases we have argued that some detachment is required to keep some engagement intact, and vice versa. In claiming that flight attendants needed to mentally detach to do their jobs. Hochschild (1983) recognised the productive work of detachment. This presents detachment as an active emotional choice; a putting aside of certain feelings in the interests of completing one's job, as we also observed. This does not mean that detachment may not, in some cases, indicate a disinterested or uncaring attitude on the part of HCAs. As suggested earlier, detachment is often described in the nursing literature as a failure to engage and an expression of burnout (Astrom et al 1990; Carmack, 1997; Morse, 1991; Omdahl & O'Donnell, 1999; Schaufeli & Bakker, 2004). We would like to augment this understanding with the view that detachment can work in the interests of engagement: in some circumstances, to detach is to engage. In this environment, if getting the job done does not always appear as the most active and inclusive form of care, this points as much to the ambiguous and conflicting demands of the medicalised/personalized discourses of health care as to the strengths or weaknesses of individual carers" (p18).<br><br>The paper also offers insight into organisational responsibility in the development of support workers;<br><br>"This brings us to the importance of the organisation taking responsibility for the emotional lives of care-givers (Kahn, 1993). At present, in the UK, HCAs are among the most poorly paid staff in health-care organisations. They receive minimal basic training, and their official job descriptions do not reflect the range of physical and emotional skills required to do the job. They are responsible for the majority of the daily personal care on these wards, yet in comparison to nurses they are given very little ongoing occupational support and are offered few opportunities to have their opinions heard. The implication of the current organisational response to HCAs is that 'non-qualified' equates to 'unskilled', and, therefore, not in need of greater financial or emotional support. Through our presentation of data, we have attempted to counter this assertion. The decisions that HCAs are required to make require great subtlety and sensitivity, as well as urgency in many cases. To negotiate the complexities of their patients' needs, dispositions and communication abilities, while also delivering the practical aspects of good care, demands a high level of emotional output and superior skills in self-regulation, which are little-recognised characteristics of the role of HCAs" (p20).<br><br>The authors offer ideas about training and supervision;<br><br>"The question of training is a complex one: the HCAs we worked alongside displayed tacit skills and knowledge which could be better recognised, reflected on and developed through education. However, when educational opportunities did arise, HCAs often found them alienating, condescending and impractical. A similar story was told in relation to supervision. When HCAs did receive supervision, it was often from the ward manager, which could mitigate against a sense of open and impartial reflection. The argument that we have presented here has highlighted the complexities of this working environment, centred on the paradox of the passive/ active patient narrative.

	In attempting to negotiate such an environment, HCAs must be given the space to reflect on the relationship between thought and action in their everyday encounters with patients.<br><br>Therefore, we suggest that developing a model of supervision along the lines of qualified nurses' clinical supervision, which is delivered by someone outside the ward, could greatly benefit HCAs" (p21).
50. Noelker & Ejaz (2005)	This paper's authors report on the challenges faced by support workers in long-term care. It calls for person-centred care approach to improve conditions for support workers and the quality of care they provide. It considers what training and education support workers want, and how this can support empowerment and advancement. In the authors' views, retention of support workers can be improved by career advancing ladders, tuition reimbursement, and education in collaboration with nursing courses.<br><br>Where workplaces demonstrate worker empowerment and person-centred care, retention and quality of care may be improved.
