# Supplementary material for: Improving skills and care standards in the support workforce for older people: a realist synthesis of workforce development interventions
Source: BMJ Open. 2016 Aug 25;6(8):e011964. doi: 10.1136/bmjopen-2016-011964 (PMC5013423; doi:10.1136/bmjopen-2016-011964)
Supplement: Supplementary additional file [file bmjopen-2016-011964supp5.pdf]

**Hypothesis 1: If workforce development is closely related to practice (cognitive and/or physical), then the intervention is more relevant and more likely to be applied**			
Sources: 4, 5, 6, 11, 13, 21,23, 26, 27, 29, 30, 31, 32, 42, 54, 56, 57, 58, 59,63, 65,71			
Study no	Supporting evidence	Impacts/ “Effectiveness”	Interpretation
4	"hands-on" patient care<br><br>Case studies, role-playing		Focuses attention on the support worker’s own work.
5<br><br>6<br><br>26<br><br>42	Staff members asked to carry out observations and to participate in fortnightly group supervision sessions. Individual support offered.<br><br>Use of residents’ biographies.<br><br>Vignettes.<br><br>Collecting biographical material stories.	Better understanding of behaviour and responses.<br><br>Increased resident’s perceptions of relationship closeness.<br><br>Learning more realistic. Empathy.<br><br>Enhanced the delivery of care.	Seeing people differently.

11	Person centered caregiving skills, experiential role-playing exercise.	Students slightly more person centered than students in the control condition.	Playing it out.<br>Copying behaviours.
13	Using focused plan to overcome a care-related problem identified within own clinical settings for nursing assistants.	Significant improvement in knowledge for nursing assistants attending the programme.	Thinking about new ways to change care.
19	Sharing feelings about what's challenging about caring for those with dementia.	Caregivers reported using more gestures, using more humour, asking more questions, and giving the choice between two options more after the workshops.	Being given time to explore own work challenges.
21	Training staff within the workplace.	Change in cohesion in staff work, and devoting more attention to residents.	Minds already in work mode.
23	In-service skills training.<br>Care planning workshops.	Interventions more likely to work if short, interactive and on the job.	Personal attention for support worker on their work.
27	Learning groups at the workplace.	More self- esteem.	Confidence building over time.
29	Groups relevant to care assistants' own practice.<br>Storytelling approach with staff sharing experiences.	Challenged task-orientated focus of care.	Personal time to explore own practice.
30	Practical assessments based on everyday situations.	Staff discussing different cases with each other might lead to deeper knowledge and understanding and encourage further learning.	Learning from peers.
31	Case conference style training.	Capturing imagination and helping individuals articulate and challenge their own mental models.	Thinking differently.

32	Role play.<br>Homework based on own practice.	Positive feelings about change in progression of getting behaviours right.	Learning what is real.
54<br><br><br><br>57<br><br>59	Debriefing through film - performance feedback/reflection on action.<br>Case studies.<br><br>Behavioural approach to care planning.	Simulation training more real, provision of safer care (knowing what to check).<br><br><br>More frequent appropriate and adequate care.<br><br>Significant increase in the time staff spent in positive interactions with residents, in terms of direct care and social contact at the end of the training.	Sense-making.
63<br><br><br><br><br><br>65	Taught component, competencies, and off the job training.<br><br><br><br><br>Training was delivered in the residential homes.	Changes to care delivery.<br>Increase sense of satisfaction and achievement.<br>Skill and knowledge development.<br>Changes related to variation in role between clinical areas.<br>Additional competencies identified.<br><br>Staff reporting in logs that they were more tolerant.<br>Caring made more enjoyable.	Immersed in own practice.
