# Supplementary material for: Improving skills and care standards in the support workforce for older people: a realist synthesis of workforce development interventions
Source: BMJ Open. 2016 Aug 25;6(8):e011964. doi: 10.1136/bmjopen-2016-011964 (PMC5013423; doi:10.1136/bmjopen-2016-011964)
Supplement: Supplementary additional file [file bmjopen-2016-011964supp6.pdf]

Additional file 6 -Set of plausible hypotheses

1. If workforce development is closely related to practice (cognitive and/or physical), then the intervention is more relevant and more likely to be applied
2. Taking staff away from practice for workforce development makes them feel more invested in, gives them more headspace, and they are more likely to feel valued by employer/organisation
3. Depending on the nature/issue/purpose of the workforce development intervention, a multi-professional approach to learning/delivery is more likely to be effective and engender cohesion
4. Where design and delivery of workforce development is seen to be credible, then support workers will engage more/ it will have more relevance
5. Where workforce development integrates personal perspective and professional perspective so that the support worker knows what's expected of them, then it may have more relevance
6. Where/if workforce development fits with the organisational strategy/philosophy then the support worker will feel more valued
7. If the focus of workforce development is on where people are coming from/starting from, and design and deliver interventions around this, they are going to be more effective
8. If workforce development is operating at more than one level (individual, team, organisation, system), then the impact is likely to be greater
9. If workforce development is appropriately targeted at individual, team, organisation, system, then it is more likely to be effective
10. Where workforce development reinforces behaviour and learning, then it is more likely to be effective
11. Where workforce development is aligned with incentives, then it is more likely to be effective
12. If there is a clearly articulated predefined theory/postulated mechanism of action about workforce development, then it is more likely to be effective
13. Where implementation features are embedded in the design and delivery of workforce development, then it is more likely to be effective
