# Supplementary material for: Improving skills and care standards in the support workforce for older people: a realist synthesis of workforce development interventions
Source: BMJ Open. 2016 Aug 25;6(8):e011964. doi: 10.1136/bmjopen-2016-011964 (PMC5013423; doi:10.1136/bmjopen-2016-011964)
Supplement: Supplementary additional file [file bmjopen-2016-011964supp7.pdf]

Additional file 7: INTERVIEW SPINE

As managers/trainers, what do you want to see for the support workforce? What are your current programmes?

1. We are interested in why some workforce development programmes resonate more with [SUPPORT WORKERS] than others. From your experience, what are the workforce development programmes that best reflect the reality of the daily work that [SUPPORT WORKERS] engage in? What is it that makes them 'real' for participants? work-based?
Prompt around characteristics: work-related; in the workplace; theory-skill mix; complex versus simple developments; short-term versus programme - seeking examples.

2. If you wanted to design a workforce development programme that really resonated with [SUPPORT WORKERS], how should this be done? Who should be involved in their design?
Prompt around people and design processes – seeking examples.

3. What teaching and learning strategies are most helpful in making workforce development 'real' for participants? What effective strategies have you come across? What else needs to be done?
Prompt around strategies and techniques (including patient stories; reflection etc., ask for examples from own experiences) – seeking examples.

4. Are there situations where the delivery of workforce development for [SUPPORT WORKERS] should include other members of the Older Person's services in health and social care?
Prompt around the relevant issues (e.g. simple versus complex workforce development) and impact (e.g. role appreciation and team-building) – seeking examples.

5. Are there good examples of where older people themselves (or carers/families/support workers) have been involved in the design and / or delivery of workforce development? Did it work? Why did this work?

6. What are the benefits and disadvantages of delivering workforce development for [SUPPORT WORKERS] in the workplace?
Prompt around benefits (e.g. real / getting experts in/translates into practice– who delivers) how this is facilitated/ and disadvantages (e.g. noise and space / difficult for time out).

7. How can we effectively build on the values and experiences that [SUPPORT WORKERS] bring to their work and role?
Prompt around previous life and work experiences; recruitment issues e.g. values; revisiting and challenging existing ways of working for those longer in the role – seeking examples.

8. What are the best ways of incentivizing [SUPPORT WORKERS] to engage in workforce development programmes? What's the tipping point? Managers' incentivisation?
Prompts around certification, accreditation, fit with career development frameworks; and financial incentives from and to the [SUPPORT WORKER] – seek examples and explanations of why incentives worked for different groups / types of [SUPPORT WORKERS].

9. What is needed at organisational level to make workforce development successful?
Prompts around organisational strategy; culture/leadership / senior buy-in; clarity and consistency of expectations across the organization; programmatic approaches versus one-off programmes – seeking examples of good organisational practice.

10. What would be the most important changes to make to workforce development programmes (yours/and in general) that would increase their impact for [SUPPORT WORKERS]? What about for health and social care organisations?
