# Supplementary material for: Improving skills and care standards in the support workforce for older people: a realist synthesis of workforce development interventions
Source: BMJ Open. 2016 Aug 25;6(8):e011964. doi: 10.1136/bmjopen-2016-011964 (PMC5013423; doi:10.1136/bmjopen-2016-011964)
Supplement: Supplementary additional file [file bmjopen-2016-011964supp8.pdf]

Additional file 8 – table of included papers

No	Authors & date	Title	Source
1	Arblaster, G., Streather, C., Hugill, L., McKenzie, M. & Missenden, J. (2004)	A training programme for healthcare support workers	*Nursing Standard*
2	Aubry, F., Etheridge, F. & Couturier, Y. (2012)	Facilitating change among nursing assistants in long term care	*Online Journal of Issues in Nursing*
3	Braun, K. L., Cheang, M. & Shigeta, D. (2005)	Increasing knowledge, skills, and empathy among direct care workers in elder care: a preliminary study of an active-learning model	*The Gerontologist*
4	Cherry, B., Marshall-Grey, P., Laurence, A., Green, A., Valadez, A., Scott-Tilley, D. & Merritt, P. (2007)	Innovative education for certified nurse aides and charge nurses	*Journal of Gerontological Nursing*
5	Clare, L., Whitaker, R., Woods, R. T., Quinn, C., Jelley, H., Hoare, Z. et al. (2013)	AwareCare: a pilot randomized controlled trial of an awareness-based staff training intervention to improve quality of life for residents with severe dementia in long-term care settings	*International Psychogeriatrics*
6	Coleman, C. & Medvene, L. (2013)	A person-centred care intervention for geriatric certified nursing assistants	*The Gerontologist*
7	Coogle, C., Parham, I., Jablonski, R. & Rachel, J. (2007)	The value of geriatric care enhancement training for direct service workers	*Gerontology and Geriatrics Education*
8	Cowan, D., Roberts, J., Fitzpatrick, J., While, A. & Baldwin, J. (2004)	The approaches to learning of support workers employed in the care home sector: an evaluation study	*Nurse Education Today*
9	Ersek, M. & Wood, B. (2008)	Development and evaluation of a nursing assistant computerised education programme	*International Journal of Palliative Nursing*
10	Goodridge, D., Johnston, P. & Thomson, M. (1997)	Impact of a nursing training program on job performance, attitudes, and relationships with residents	*Educational Gerontology*

11	Grosch, K., Medvene, L. & Wolcott, H. (2008)	Person-centred care-giving instruction for geriatric nursing assistant students	*Journal of Gerontological Nursing*
12	Hegeman, C. (2003)	Peer mentoring of nursing home CNAs: a way to create a culture of caring	*Journal of Social Work in Long -term care*
13	Lerner, N., , Resnick, B., Galik, E. & Gunther Russ, K. (2010)	Advanced nursing assistant education program	*The Journal of Continuing Education in Nursing*
14	Morgan, J. & Conrad, T. (2008)	A mixed-method evaluation of a workforce development intervention for nursing assistants in nursing homes: the case of WIN A STEP UP	*The Gerontologist*
15	Nelson, S. & Wild, D. (2009)	The forgotten sector: workforce development in residential care for older people	*Nursing and Residential Care*
16	Noelker, L., Ejaz, F., Menne, H. & Jones, J. (2006)	The Impact of stress and support on nursing assistant satisfaction with supervision	*Journal of Applied Gerontology*
17	Noelker, L.,Ejaz, F., Menne, H. & Bagaka, J. (2009)	Factors affecting frontline workers' satisfaction with supervision	*Journal of Aging and Health*
18	Parks, S. M., Haines, C., Foreman, D., McKinstry, E. & Maxwell, T. L. (2005)	Evaluation of an educational program for long term care nursing assistants	*Journal of the American Medical Directors Association*
19	Passalacqua, S. & Harwood, J. (2012)	VIPS communication skills training for paraprofessional dementia caregivers: an intervention to increase person-centered dementia care	*Clinical Gerontologist*
20	Petterson, I-L., Donnersvard, H., Lagerstrom, M. & Toomingas, A. (2006)	Evaluation of an intervention programme based on empowerment for eldercare nursing staff	*Work and Stress*
21	Ron, P. & Lowenstein, A. (2002)	In service training of professional and para-professional staff in institutions for the aged	*Educational Gerontology*
22	Ruckdeschel, K. & Van Haitsma, K. (2004)	A workshop for nursing home staff	*Gerontology and Geriatrics Education*
23	Stevens, A. B., Hochhalter, A. K., Hyer, L. & Intrieri, R. C. (2006)	Meeting the needs of nursing home residents and staff	*Geropsychological Interventions in Long-Term Care*

24	Stevens-Roseman, E. & Leung, P. (2004)	Enhancing attitudes, knowledge, and skills of paraprofessional service providers in elder care settings	*Gerontology and Geriatrics Education*
25	Thomson, M. & Burke, K. (1998)	A nursing assistant training program in a long term care setting	*Gerontology and Geriatrics Education*
26	Tisher, T., Dean, S. & Tisher, M. (2009)	Aged care residential facility and family interface: a training program for staff	*Clinical Gerontologist*
27	Wadenstein, B., Engstrom, M. & Haggstrom, E. (2009)	Public nursing home staffs experience of participating in an intervention aimed at enhancing their self-esteem	*Journal of Nursing Management*
28	White, D. & Cadiz, D. (2013)	Efficacy of work-based training for direct care workers in assisted living	*Journal of Aging and Social Policy*
29	Hockley, J. (2014)	Learning, support and communication for staff in care homes: outcomes of reflective debriefing groups in two care homes to enhance end-of-life care	*International Journal of Older people's Nursing*
30	Nilsson, A., Andren, M. & Engstrom, M. (2014)	E-assessment of prior learning: a pilot study of interactive assessment of staff with no formal education who are working in Swedish elderly care	*BMC Geriatrics*
31	Anderson, R.,Ammarell, N., Bailey, D., Colón-Emeric, C., Corazzini, K., Lillie, M. et al. (2005)	Nurse assistant mental models, sense-making, care actions, and consequences for nursing home residents	*Qualitative Health Research*
32	Boettcher, I., Kemeny, B., DeShon, R., Stevens, A. (2004)	A system to develop staff behaviours for person-centered care	*Alzheimer's Care Quarterly*
33	McGilton, K., O'Brien-Pallas, L., Darlington, G., Evans, M., Wynn, F. & Pringle, D. (2003)	Effects of a relationship-enhancing program of care on outcomes	*Clinical Scholarship*
34	McCormack, B., Dewing, L., Breslin, L., Coyne-Nevin, A., Kennedy, K., Manning, M. et al. (2010)	Developing person-centred practice: nursing outcomes arising from changes to the care environment in residential settings for older people	*International Journal of Older People's Nursing*

35	McCormack, B. & Wright, J. (1999)	Achieving dignified care for older people through practice development: a systematic approach	*Nursing Times Research*
36	Yalden, J., McCormack, B., O'Connor, M. & Hardy, S. (1999)	Transforming end of life care using practice development : an arts-informed approach in residential aged care	*International Practice Development Journal*
37	Webster, J., Coats, E. & Noble, G. (2009)	Enabling dignity in care through practice development with older people	*Practice Development in Health Care*
38	Howatson-Jones, L., Thurgate, C., Graber, M., Harnett, C., Thompson, J. & Jordan, D. (2012)	Biographical learning: a process for promoting person-centeredness in nursing	*International Practice Development Journal*
39	Manley, K., Sanders, K., Cardiff, S. & Webster, J. (2011)	Effective workplace culture: the attributes, enabling factors and consequences of a new concept	*International Practice Development Journal*
40	Health Service Executive. (2010)	The implementation of a model of person-centred practice in older persons setting	*Health Service Executive*
41	McCormack, B. (2003)	A conceptual framework for person-centred practice with older people	*International Journal of Nursing Practice*
42	Clarke, A. C., Hanson, E. J. & Ross, H. (2003)	Seeing the person behind the patient: enhancing the care of older people using a biographical approach	*Journal of Clinical Nursing*
43	Medvene, L. & Lann-Wolcott, H. (2009)	An exploratory study of nurse aides' communication behaviours: giving "positive regard" as a strategy	*International Journal of Older people Nursing*
44	Brown, J., Nolan, M., Davies, S., Nolan, J. & Keady, J. (2007)	Transforming students' views of gerontological nursing: Realising the potential of "enriched" environments of learning and care: a multi-method longitudinal study	*International Journal of Nursing Studies*
45	McLellan, H., Bateman, H. & Bailey, P. (2005)	The place of 360 degree appraisal within a team approach to professional development	*Journal of Interprofessional care*
46	Parry, R. & Vass, C. (1997)	Training and assessment of physiotherapy assistants	*Physiotherapy*

47	Graber, D. R., Mitcham, M. D., Coker-Bolt, P., Wise, H. H., Jacques, P., Edlund, B. & Annan-Coultas, D. (2012)	The caring professionals program: educational approaches that integrate caring attitudes and empathic behaviours into health professions education	*Journal of Allied Health*
48	Vail, L., Bosley, S., Petrova, M. & Dale, J (2011)	Healthcare assistants in general practice: a qualitative study of their experiences	*Primary Health Care Research*
49	Bailey, S., Scales, K., Lloyd, J., Schneider, J. & Jones, R. (2013)	The emotional labour of healthcare assistants in inpatient dementia care	*Ageing and Society*
50	Noelker, L. S. & Ejaz, F. K. (2005)	Training direct-care workers for person-centered care	*Public Policy & Aging Report*
51	Welsh, J. D. & Szabo, G. B. (2011)	Teaching nursing assistant students about aphasia and communication	*Seminars in Speech and Language*
52	Ryan, T., Nolan, M., Enderby, P. & Reid, D. (2004)	Part of the family: sources of job satisfaction amongst a group of community –based dementia care workers	*Health and Social Care in the Community*
53	Gethin-Jones, S. (2013)	Focus on the micro-relationship of care	*British Journal of Healthcare Assistants*
54	McKenzie-Smith, M. (2013)	Simulation-based education in support of HCA development	*British Journal of Healthcare Assistants*
55	Godfrey, A. (2000)	What impact does training have on the care received by older people in residential homes?	*Social Work Education*
56	McCallion, P., Toseland, R. W., Lacey, D. & Banks, S. (1999)	Educating nursing assistants to communicate more effectively with nursing home residents with dementia	*The Gerontologist*
57	Smith, B., Kerse, N. & Parsons, M. (2005)	Quality of residential care for older people: does education for healthcare assistants make a difference?	*The New Zealand Medical Journal*
58	Lewis, R., Kelly, S., Whitfield, M., Mckenzie-Smith, M. & Strachan, A. (2013)	An evaluation of a simulation-based educational programme to equip HCAs with the necessary non-technical skills to undertake their role safely and effectively,	*Yorkshire & Humber Strategic Health Authority*

		specifically in relation to the measurement of vital signs	
59	Proctor, R., Stratton Powell, H., Burns, A., Tarrier, N., Reeves, D., Emerson, E. & Hatton, C. (1998)	An observational study to evaluate the impact of a specialist outreach team on the quality of care in nursing and residential homes	*Aging & Mental Health*
60	Burgio, L. D., Allen-Burge, R., Roth D. L., Bourgeois, M. S., Dijkstra, K., Gerstle, J. et al (2001)	Come talk with me: improving communication between nursing assistants and nursing home residents during care routines	*The Gerontologist*
61	Bourgeois, M., Dijkstra, K., Burgio, L. & Allen, R. (2004)	Communication skills training for nursing aides of residents with dementia	*Clinical Gerontologist*
62	Latta, L & Ross, J (2010)	Exploring the impact of palliative care education for care assistants employed in residential aged care facilities in Otago, New Zealand	*SITES*
63	Hancock, H., Campbell, S., Ramprogus, V. & Kilgour, J (2005)	Role development in healthcare assistants: the impact of education on practice	*Journal of Evaluation in Clinical Practice*
64	Chapman, A. & Law, S. (2009)	Bridging the gap: an innovative dementia learning program for healthcare assistants in hospital wards using facilitator-led discussions	*International Psychogeriatrics*
65	Moxon, S., Lyne, K., Sinclair, I., Young, P. & Kirk, C. (2001)	Mental health in residential homes: a role for care staff	*Ageing and Society*
66	Kuske, B., Luck, T., Hanns, S., Matschinger, H., Angermyer, M., Behrens, J. & Riedel-Heller, S. (2009)	Training in dementia care: a cluster-randomised controlled trial of a training program for nursing home staff in Germany	*International Psychogeriatrics*
67	Bryan, K., Axelrod, L., Maxim, J., Bell, L. & Jordan. L. (2002)	Working with older people with communication difficulties: an evaluation of care worker training	*Aging and Mental Health*
68 ** from policing*	Sutherland, J. (2014)	The PCSO review- an evaluation of the role, value and establishment of police	*Cambridgeshire Constabulary*

		community support officers within Cambridgeshire constabulary	
69* *from policing*	O'Neill, M. (2014)	PCSOS as the paraprofessionals of policing	*Leverhulme Trust*
70* *from policing*	Cooper, C., Anscombe, J., Avenell, J., McLean, F. & Morris, J. (2006)	A national evaluation of community support officers	*Home Office Research Study 297*
71* *from education*	Farrell, P., Balshaw, M. & Polat, F. (1999)	The management, role and training of learning support assistants	*Department for Education and Employment*
72* *from education*	Jolly, A. & Evans, S. (2005)	Teacher assistants move to the front of the class: job-embedded learning pays off in student achievement	*The Journal of Staff Development*
73* *from education*	Groom, B. (2006)	Building relationships for learning: the developing role of the teaching assistant	*Support for Learning*
74* *from education*	Collins, J. & Simco, N. (2006)	Teaching assistants reflect: the way forward?	*Reflective Practice: International and Multidisciplinary Perspectives*
75 * *from education*	Righter, B. (1987)	Training for teaching assistants	*Engineering Education*
76	Santo Pietro, M. (2002)	Training nursing assistants to communicate effectively with persons with Alzheimer's Disease: a call for action	*Alzheimer's Care Quarterly*
